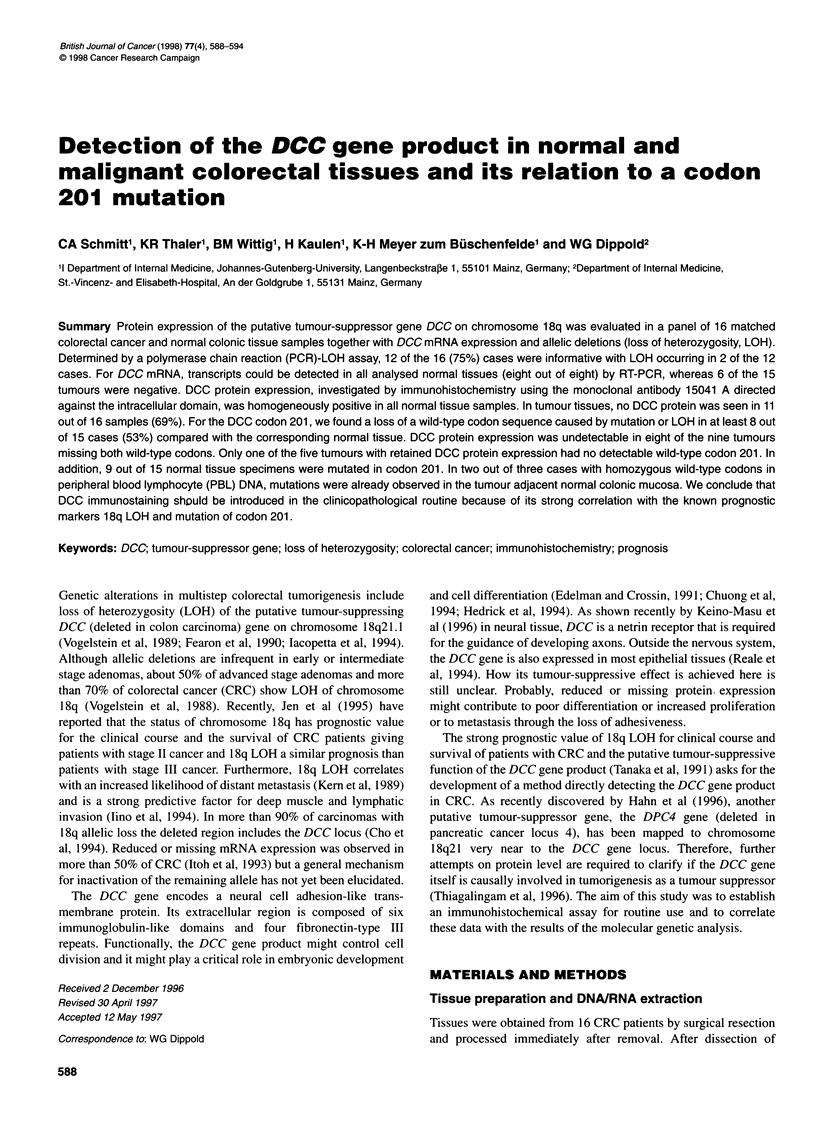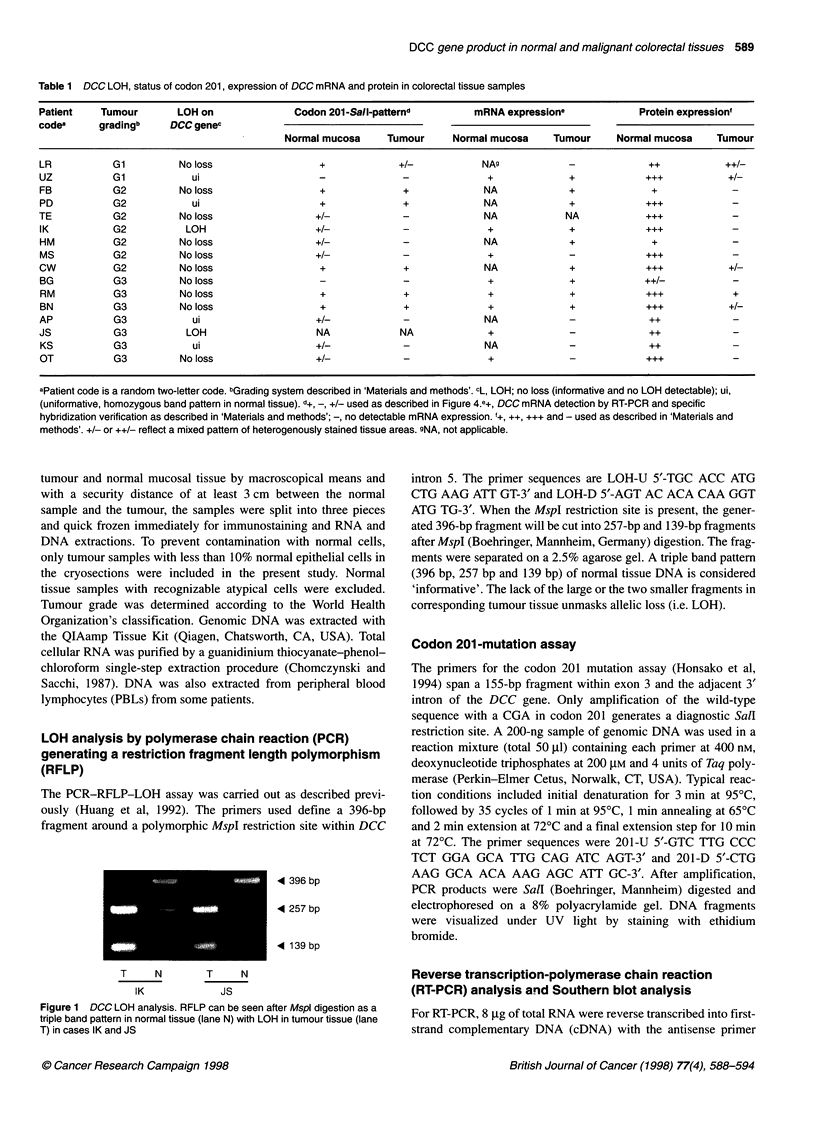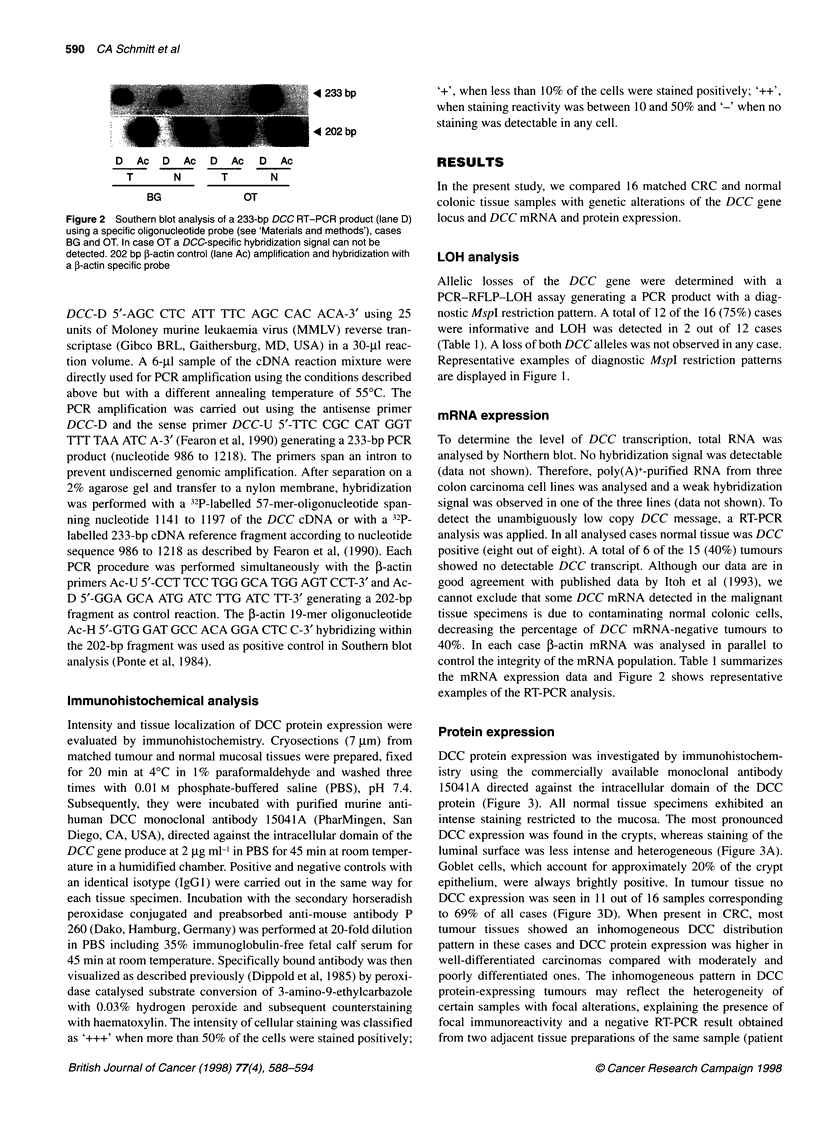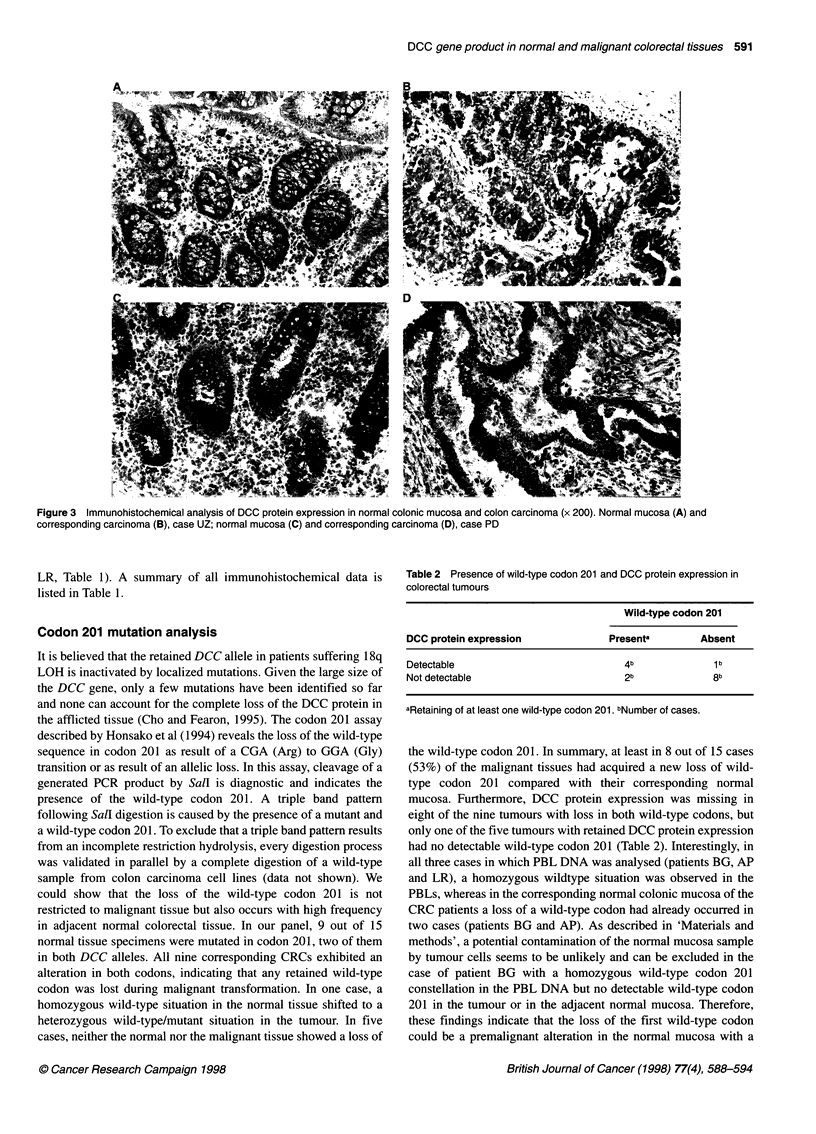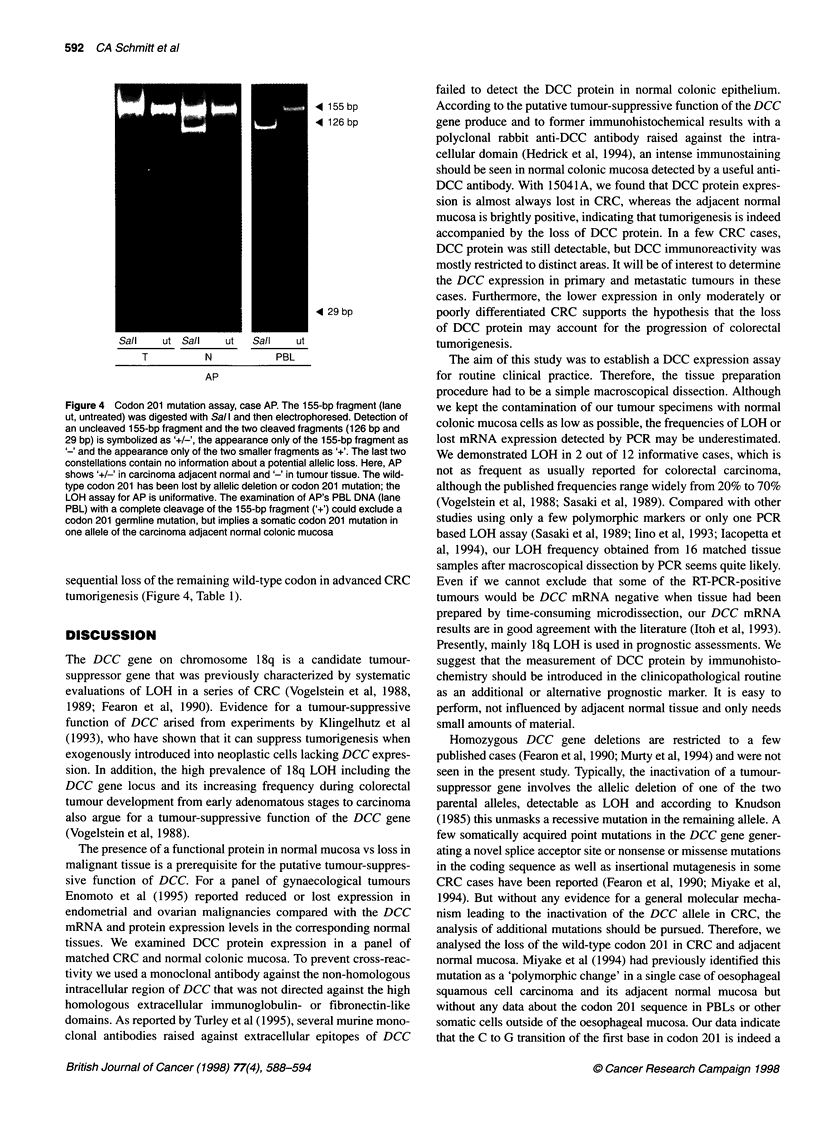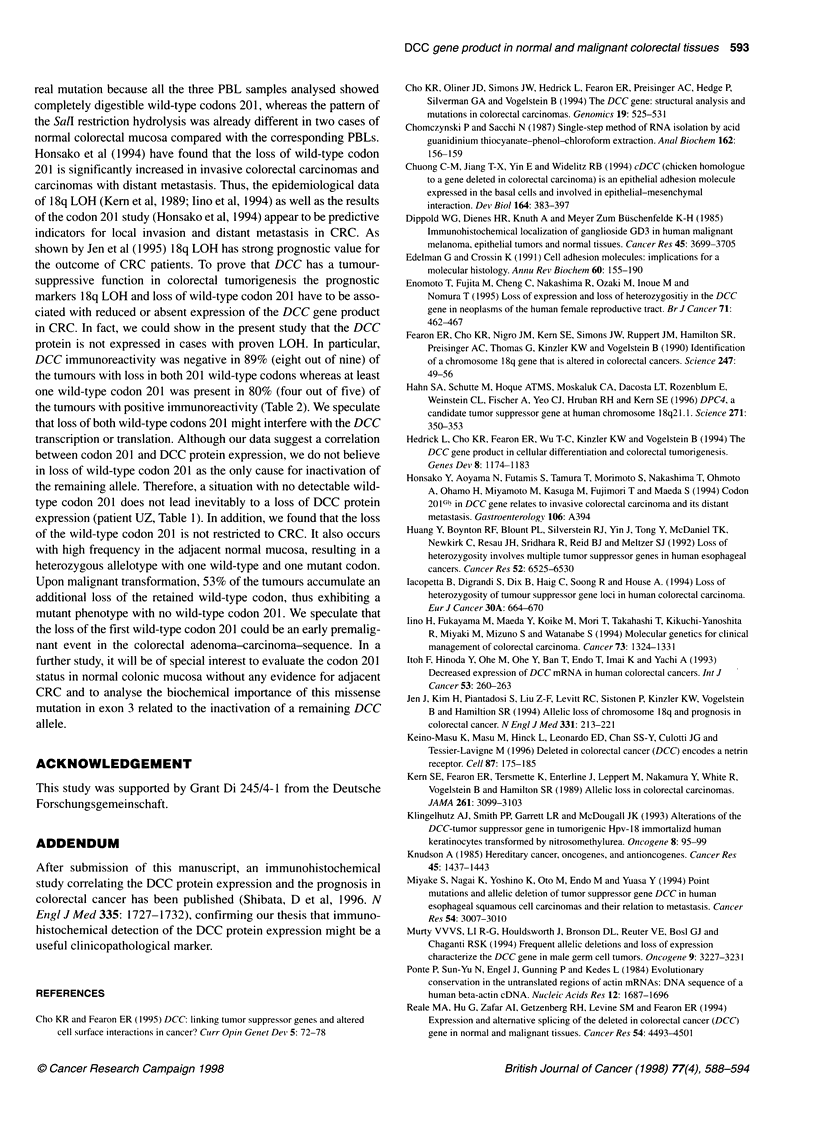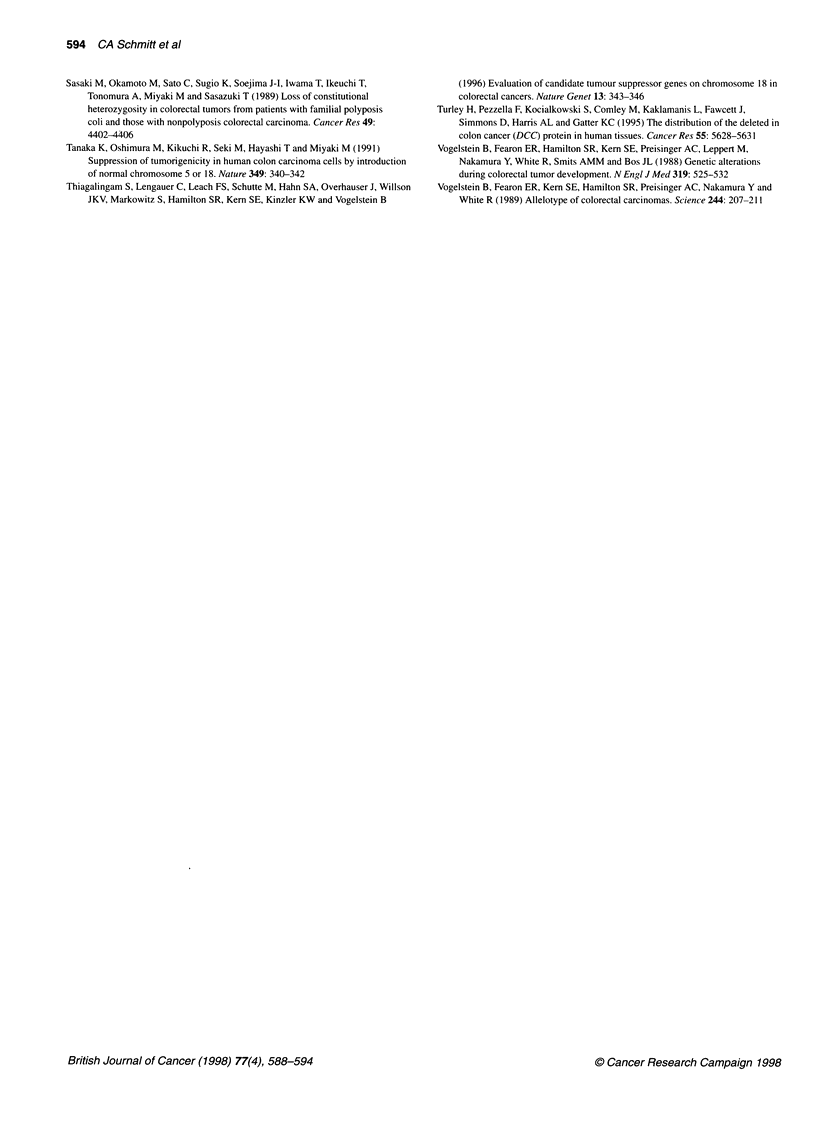# Detection of the DCC gene product in normal and malignant colorectal tissues and its relation to a codon 201 mutation.

**DOI:** 10.1038/bjc.1998.95

**Published:** 1998-02

**Authors:** C. A. Schmitt, K. R. Thaler, B. M. Wittig, H. Kaulen, K. H. Meyer zum BÃ¼schenfelde, W. G. Dippold

**Affiliations:** I Department of Internal Medicine, Johannes-Gutenberg-University, Mainz, Germany.

## Abstract

**Images:**


British Journal of Cancer (1998) 77(4), 588–594
© 1998 Cancer Research Campaign

### Detection of the *DCC* gene product in normal and malignant colorectal tissues and its relation to a codon 201 mutation

**CA Schmitt[1], KR Thaler[1], BM Wittig[1], H Kaulen[1], K-H Meyer zum Büschenfelde[1] and WG Dippold[2]**

[1]I Department of Internal Medicine, Johannes-Gutenberg-University, Langenbeckstraße 1, 55101 Mainz, Germany; [2]Department of Internal Medicine, St.-Vincenz- and Elisabeth-Hospital, An der Goldgrube 1, 55131 Mainz, Germany

**Summary** Protein expression of the putative tumour-suppressor gene *DCC* on chromosome 18q was evaluated in a panel of 16 matched colorectal cancer and normal colonic tissue samples together with *DCC* mRNA expression and allelic deletions (loss of heterozygosity, LOH). Determined by a polymerase chain reaction (PCR)-LOH assay, 12 of the 16 (75%) cases were informative with LOH occurring in 2 of the 12 cases. For *DCC* mRNA, transcripts could be detected in all analysed normal tissues (eight out of eight) by RT-PCR, whereas 6 of the 15 tumours were negative. DCC protein expression, investigated by immunohistochemistry using the monoclonal antibody 15041 A directed against the intracellular domain, was homogeneously positive in all normal tissue samples. In tumour tissues, no DCC protein was seen in 11 out of 16 samples (69%). For the DCC codon 201, we found a loss of a wild-type codon sequence caused by mutation or LOH in at least 8 out of 15 cases (53%) compared with the corresponding normal tissue. DCC protein expression was undetectable in eight of the nine tumours missing both wild-type codons. Only one of the five tumours with retained DCC protein expression had no detectable wild-type codon 201. In addition, 9 out of 15 normal tissue specimens were mutated in codon 201. In two out of three cases with homozygous wild-type codons in peripheral blood lymphocyte (PBL) DNA, mutations were already observed in the tumour adjacent normal colonic mucosa. We conclude that DCC immunostaining should be introduced in the clinicopathological routine because of its strong correlation with the known prognostic markers 18q LOH and mutation of codon 201.

**Keywords:** *DCC*; tumour-suppressor gene; loss of heterozygosity; colorectal cancer; immunohistochemistry; prognosis

Genetic alterations in multistep colorectal tumorigenesis include loss of heterozygosity (LOH) of the putative tumour-suppressing *DCC* (deleted in colon carcinoma) gene on chromosome 18q21.1 (Vogelstein et al, 1989; Fearon et al, 1990; Iacopetta et al, 1994). Although allelic deletions are infrequent in early or intermediate stage adenomas, about 50% of advanced stage adenomas and more than 70% of colorectal cancer (CRC) show LOH of chromosome 18q (Vogelstein et al, 1988). Recently, Jen et al (1995) have reported that the status of chromosome 18q has prognostic value for the clinical course and the survival of CRC patients giving patients with stage II cancer and 18q LOH a similar prognosis than patients with stage III cancer. Furthermore, 18q LOH correlates with an increased likelihood of distant metastasis (Kern et al, 1989) and is a strong predictive factor for deep muscle and lymphatic invasion (Iino et al, 1994). In more than 90% of carcinomas with 18q allelic loss the deleted region includes the *DCC* locus (Cho et al, 1994). Reduced or missing mRNA expression was observed in more than 50% of CRC (Itoh et al, 1993) but a general mechanism for inactivation of the remaining allele has not yet been elucidated.

The *DCC* gene encodes a neural cell adhesion-like transmembrane protein. Its extracellular region is composed of six immunoglobulin-like domains and four fibronectin-type III repeats. Functionally, the *DCC* gene product might control cell division and it might play a critical role in embryonic development and cell differentiation (Edelman and Crossin, 1991; Chuong et al, 1994; Hedrick et al, 1994). As shown recently by Keino-Masu et al (1996) in neural tissue, *DCC* is a netrin receptor that is required for the guidance of developing axons. Outside the nervous system, the *DCC* gene is also expressed in most epithelial tissues (Reale et al, 1994). How its tumour-suppressive effect is achieved here is still unclear. Probably, reduced or missing protein expression might contribute to poor differentiation or increased proliferation or to metastasis through the loss of adhesiveness.

The strong prognostic value of 18q LOH for clinical course and survival of patients with CRC and the putative tumour-suppressive function of the *DCC* gene product (Tanaka et al, 1991) asks for the development of a method directly detecting the *DCC* gene product in CRC. As recently discovered by Hahn et al (1996), another putative tumour-suppressor gene, the *DPC4* gene (deleted in pancreatic cancer locus 4), has been mapped to chromosome 18q21 very near to the *DCC* gene locus. Therefore, further attempts on protein level are required to clarify if the *DCC* gene itself is causally involved in tumorigenesis as a tumour suppressor (Thiagalingam et al, 1996). The aim of this study was to establish an immunohistochemical assay for routine use and to correlate these data with the results of the molecular genetic analysis.

*Received 2 December 1996*
*Revised 30 April 1997*
*Accepted 12 May 1997*

*Correspondence to*: WG Dippold

### MATERIALS AND METHODS

### Tissue preparation and DNA/RNA extraction

Tissues were obtained from 16 CRC patients by surgical resection and processed immediately after removal. After dissection of

**Table 1** *DCC* LOH, status of codon 201, expression of *DCC* mRNA and protein in colorectal tissue samples

| Patient code[a] | Tumour grading[b] | LOH on *DCC* gene[c] | Codon 201-*Sal*I-pattern[d] | | mRNA expression[e] | | Protein expression[f] | |
|---|---|---|---|---|---|---|---|---|
| | | | Normal mucosa | Tumour | Normal mucosa | Tumour | Normal mucosa | Tumour |
| LR | G1 | No loss | + | +/– | NA[g] | – | ++ | ++/– |
| UZ | G1 | ui | – | – | + | + | +++ | +/– |
| FB | G2 | No loss | + | + | NA | + | + | – |
| PD | G2 | ui | + | + | NA | + | +++ | – |
| TE | G2 | No loss | +/– | – | NA | NA | +++ | – |
| IK | G2 | LOH | +/– | – | + | + | +++ | – |
| HM | G2 | No loss | +/– | – | NA | + | + | – |
| MS | G2 | No loss | +/– | – | + | – | +++ | – |
| CW | G2 | No loss | + | + | NA | + | +++ | +/– |
| BG | G3 | No loss | – | – | + | + | ++/– | – |
| RM | G3 | No loss | + | + | + | + | +++ | + |
| BN | G3 | No loss | + | + | + | + | +++ | +/– |
| AP | G3 | ui | +/– | – | NA | – | ++ | – |
| JS | G3 | LOH | NA | NA | + | – | ++ | – |
| KS | G3 | ui | +/– | – | NA | – | ++ | – |
| OT | G3 | No loss | +/– | – | + | – | +++ | – |

[a]Patient code is a random two-letter code. [b]Grading system described in 'Materials and methods'. [c]L, LOH; no loss (informative and no LOH detectable); ui, (uniformative, homozygous band pattern in normal tissue). [d]+, –, +/– used as described in Figure 4.[e]+, *DCC* mRNA detection by RT-PCR and specific hybridization verification as described in 'Materials and methods'; –, no detectable mRNA expression. [f]+, ++, +++ and – used as described in 'Materials and methods'. +/– or ++/– reflect a mixed pattern of heterogenously stained tissue areas. [g]NA, not applicable.

tumour and normal mucosal tissue by macroscopical means and with a security distance of at least 3 cm between the normal sample and the tumour, the samples were split into three pieces and quick frozen immediately for immunostaining and RNA and DNA extractions. To prevent contamination with normal cells, only tumour samples with less than 10% normal epithelial cells in the cryosections were included in the present study. Normal tissue samples with recognizable atypical cells were excluded. Tumour grade was determined according to the World Health Organization's classification. Genomic DNA was extracted with the QIAamp Tissue Kit (Qiagen, Chatsworth, CA, USA). Total cellular RNA was purified by a guanidinium thiocyanate–phenol–chloroform single-step extraction procedure (Chomczynski and Sacchi, 1987). DNA was also extracted from peripheral blood lymphocytes (PBLs) from some patients.

### LOH analysis by polymerase chain reaction (PCR) generating a restriction fragment length polymorphism (RFLP)

The PCR–RFLP–LOH assay was carried out as described previously (Huang et al, 1992). The primers used define a 396-bp fragment around a polymorphic *Msp*I restriction site within *DCC* intron 5. The primer sequences are LOH-U 5′-TGC ACC ATG CTG AAG ATT GT-3′ and LOH-D 5′-AGT AC ACA CAA GGT ATG TG-3′. When the *Msp*I restriction site is present, the generated 396-bp fragment will be cut into 257-bp and 139-bp fragments after *Msp*I (Boehringer, Mannheim, Germany) digestion. The fragments were separated on a 2.5% agarose gel. A triple band pattern (396 bp, 257 bp and 139 bp) of normal tissue DNA is considered 'informative'. The lack of the large or the two smaller fragments in corresponding tumour tissue unmasks allelic loss (i.e. LOH).

**Figure 1** *DCC* LOH analysis. RFLP can be seen after *Msp*I digestion as a triple band pattern in normal tissue (lane N) with LOH in tumour tissue (lane T) in cases IK and JS

### Codon 201-mutation assay

The primers for the codon 201 mutation assay (Honsako et al, 1994) span a 155-bp fragment within exon 3 and the adjacent 3′ intron of the *DCC* gene. Only amplification of the wild-type sequence with a CGA in codon 201 generates a diagnostic *Sal*I restriction site. A 200-ng sample of genomic DNA was used in a reaction mixture (total 50 μl) containing each primer at 400 nM, deoxynucleotide triphosphates at 200 μM and 4 units of *Taq* polymerase (Perkin–Elmer Cetus, Norwalk, CT, USA). Typical reaction conditions included initial denaturation for 3 min at 95°C, followed by 35 cycles of 1 min at 95°C, 1 min annealing at 65°C and 2 min extension at 72°C and a final extension step for 10 min at 72°C. The primer sequences were 201-U 5′-GTC TTG CCC TCT GGA GCA TTG CAG ATC AGT-3′ and 201-D 5′-CTG AAG GCA ACA AAG AGC ATT GC-3′. After amplification, PCR products were *Sal*I (Boehringer, Mannheim) digested and electrophoresed on a 8% polyacrylamide gel. DNA fragments were visualized under UV light by staining with ethidium bromide.

### Reverse transcription-polymerase chain reaction (RT-PCR) analysis and Southern blot analysis

For RT-PCR, 8 μg of total RNA were reverse transcribed into first-strand complementary DNA (cDNA) with the antisense primer

© Cancer Research Campaign 1998 

**Figure 2** Southern blot analysis of a 233-bp *DCC* RT–PCR product (lane D) using a specific oligonucleotide probe (see 'Materials and methods'), cases BG and OT. In case OT a *DCC*-specific hybridization signal can not be detected. 202 bp β-actin control (lane Ac) amplification and hybridization with a β-actin specific probe

*DCC*-D 5′-AGC CTC ATT TTC AGC CAC ACA-3′ using 25 units of Moloney murine leukaemia virus (MMLV) reverse transcriptase (Gibco BRL, Gaithersburg, MD, USA) in a 30-μl reaction volume. A 6-μl sample of the cDNA reaction mixture were directly used for PCR amplification using the conditions described above but with a different annealing temperature of 55°C. The PCR amplification was carried out using the antisense primer *DCC*-D and the sense primer *DCC*-U 5′-TTC CGC CAT GGT TTT TAA ATC A-3′ (Fearon et al, 1990) generating a 233-bp PCR product (nucleotide 986 to 1218). The primers span an intron to prevent undiscerned genomic amplification. After separation on a 2% agarose gel and transfer to a nylon membrane, hybridization was performed with a $^{32}$P-labelled 57-mer-oligonucleotide spanning nucleotide 1141 to 1197 of the *DCC* cDNA or with a $^{32}$P-labelled 233-bp cDNA reference fragment according to nucleotide sequence 986 to 1218 as described by Fearon et al, (1990). Each PCR procedure was performed simultaneously with the β-actin primers Ac-U 5′-CCT TCC TGG GCA TGG AGT CCT-3′ and Ac-D 5′-GGA GCA ATG ATC TTG ATC TT-3′ generating a 202-bp fragment as control reaction. The β-actin 19-mer oligonucleotide Ac-H 5′-GTG GAT GCC ACA GGA CTC C-3′ hybridizing within the 202-bp fragment was used as positive control in Southern blot analysis (Ponte et al, 1984).

### Immunohistochemical analysis

Intensity and tissue localization of DCC protein expression were evaluated by immunohistochemistry. Cryosections (7 μm) from matched tumour and normal mucosal tissues were prepared, fixed for 20 min at 4°C in 1% paraformaldehyde and washed three times with 0.01 M phosphate-buffered saline (PBS), pH 7.4. Subsequently, they were incubated with purified murine anti-human DCC monoclonal antibody 15041A (PharMingen, San Diego, CA, USA), directed against the intracellular domain of the *DCC* gene produce at 2 μg ml$^{-1}$ in PBS for 45 min at room temperature in a humidified chamber. Positive and negative controls with an identical isotype (IgG1) were carried out in the same way for each tissue specimen. Incubation with the secondary horseradish peroxidase conjugated and preabsorbed anti-mouse antibody P 260 (Dako, Hamburg, Germany) was performed at 20-fold dilution in PBS including 35% immunoglobulin-free fetal calf serum for 45 min at room temperature. Specifically bound antibody was then visualized as described previously (Dippold et al, 1985) by peroxidase catalysed substrate conversion of 3-amino-9-ethylcarbazole with 0.03% hydrogen peroxide and subsequent counterstaining with haematoxylin. The intensity of cellular staining was classified as '+++' when more than 50% of the cells were stained positively; '+', when less than 10% of the cells were stained positively; '++', when staining reactivity was between 10 and 50% and '–' when no staining was detectable in any cell.

### RESULTS

In the present study, we compared 16 matched CRC and normal colonic tissue samples with genetic alterations of the *DCC* gene locus and *DCC* mRNA and protein expression.

### LOH analysis

Allelic losses of the *DCC* gene were determined with a PCR–RFLP–LOH assay generating a PCR product with a diagnostic *Msp*I restriction pattern. A total of 12 of the 16 (75%) cases were informative and LOH was detected in 2 out of 12 cases (Table 1). A loss of both *DCC* alleles was not observed in any case. Representative examples of diagnostic *Msp*I restriction patterns are displayed in Figure 1.

### mRNA expression

To determine the level of *DCC* transcription, total RNA was analysed by Northern blot. No hybridization signal was detectable (data not shown). Therefore, poly(A)$^+$-purified RNA from three colon carcinoma cell lines was analysed and a weak hybridization signal was observed in one of the three lines (data not shown). To detect the unambiguously low copy *DCC* message, a RT-PCR analysis was applied. In all analysed cases normal tissue was *DCC* positive (eight out of eight). A total of 6 of the 15 (40%) tumours showed no detectable *DCC* transcript. Although our data are in good agreement with published data by Itoh et al (1993), we cannot exclude that some *DCC* mRNA detected in the malignant tissue specimens is due to contaminating normal colonic cells, decreasing the percentage of *DCC* mRNA-negative tumours to 40%. In each case β-actin mRNA was analysed in parallel to control the integrity of the mRNA population. Table 1 summarizes the mRNA expression data and Figure 2 shows representative examples of the RT-PCR analysis.

### Protein expression

DCC protein expression was investigated by immunohistochemistry using the commercially available monoclonal antibody 15041A directed against the intracellular domain of the DCC protein (Figure 3). All normal tissue specimens exhibited an intense staining restricted to the mucosa. The most pronounced DCC expression was found in the crypts, whereas staining of the luminal surface was less intense and heterogeneous (Figure 3A). Goblet cells, which account for approximately 20% of the crypt epithelium, were always brightly positive. In tumour tissue no DCC expression was seen in 11 out of 16 samples corresponding to 69% of all cases (Figure 3D). When present in CRC, most tumour tissues showed an inhomogeneous DCC distribution pattern in these cases and DCC protein expression was higher in well-differentiated carcinomas compared with moderately and poorly differentiated ones. The inhomogeneous pattern in DCC protein-expressing tumours may reflect the heterogeneity of certain samples with focal alterations, explaining the presence of focal immunoreactivity and a negative RT-PCR result obtained from two adjacent tissue preparations of the same sample (patient

 © Cancer Research Campaign 1998

**Figure 3** Immunohistochemical analysis of DCC protein expression in normal colonic mucosa and colon carcinoma (× 200). Normal mucosa (**A**) and corresponding carcinoma (**B**), case UZ; normal mucosa (**C**) and corresponding carcinoma (**D**), case PD

LR, Table 1). A summary of all immunohistochemical data is listed in Table 1.

### Codon 201 mutation analysis

It is believed that the retained *DCC* allele in patients suffering 18q LOH is inactivated by localized mutations. Given the large size of the *DCC* gene, only a few mutations have been identified so far and none can account for the complete loss of the DCC protein in the afflicted tissue (Cho and Fearon, 1995). The codon 201 assay described by Honsako et al (1994) reveals the loss of the wild-type sequence in codon 201 as result of a CGA (Arg) to GGA (Gly) transition or as result of an allelic loss. In this assay, cleavage of a generated PCR product by *Sal*I is diagnostic and indicates the presence of the wild-type codon 201. A triple band pattern following *Sal*I digestion is caused by the presence of a mutant and a wild-type codon 201. To exclude that a triple band pattern results from an incomplete restriction hydrolysis, every digestion process was validated in parallel by a complete digestion of a wild-type sample from colon carcinoma cell lines (data not shown). We could show that the loss of the wild-type codon 201 is not restricted to malignant tissue but also occurs with high frequency in adjacent normal colorectal tissue. In our panel, 9 out of 15 normal tissue specimens were mutated in codon 201, two of them in both *DCC* alleles. All nine corresponding CRCs exhibited an alteration in both codons, indicating that any retained wild-type codon was lost during malignant transformation. In one case, a homozygous wild-type situation in the normal tissue shifted to a heterozygous wild-type/mutant situation in the tumour. In five cases, neither the normal nor the malignant tissue showed a loss of

**Table 2** Presence of wild-type codon 201 and DCC protein expression in colorectal tumours

| | Wild-type codon 201 | |
|---|---|---|
| **DCC protein expression** | **Present**[a] | **Absent** |
| Detectable | 4[b] | 1[b] |
| Not detectable | 2[b] | 8[b] |

[a]Retaining of at least one wild-type codon 201. [b]Number of cases.

the wild-type codon 201. In summary, at least in 8 out of 15 cases (53%) of the malignant tissues had acquired a new loss of wild-type codon 201 compared with their corresponding normal mucosa. Furthermore, DCC protein expression was missing in eight of the nine tumours with loss in both wild-type codons, but only one of the five tumours with retained DCC protein expression had no detectable wild-type codon 201 (Table 2). Interestingly, in all three cases in which PBL DNA was analysed (patients BG, AP and LR), a homozygous wildtype situation was observed in the PBLs, whereas in the corresponding normal colonic mucosa of the CRC patients a loss of a wild-type codon had already occurred in two cases (patients BG and AP). As described in 'Materials and methods', a potential contamination of the normal mucosa sample by tumour cells seems to be unlikely and can be excluded in the case of patient BG with a homozygous wild-type codon 201 constellation in the PBL DNA but no detectable wild-type codon 201 in the tumour or in the adjacent normal mucosa. Therefore, these findings indicate that the loss of the first wild-type codon could be a premalignant alteration in the normal mucosa with a

© Cancer Research Campaign 1998

**Figure 4** Codon 201 mutation assay, case AP. The 155-bp fragment (lane ut, untreated) was digested with *Sal* I and then electrophoresed. Detection of an uncleaved 155-bp fragment and the two cleaved fragments (126 bp and 29 bp) is symbolized as '+/–', the appearance only of the 155-bp fragment as '–' and the appearance only of the two smaller fragments as '+'. The last two constellations contain no information about a potential allelic loss. Here, AP shows '+/–' in carcinoma adjacent normal and '–' in tumour tissue. The wild-type codon 201 has been lost by allelic deletion or codon 201 mutation; the LOH assay for AP is uniformative. The examination of AP's PBL DNA (lane PBL) with a complete cleavage of the 155-bp fragment ('+') could exclude a codon 201 germline mutation, but implies a somatic codon 201 mutation in one allele of the carcinoma adjacent normal colonic mucosa sequential loss of the remaining wild-type codon in advanced CRC tumorigenesis (Figure 4, Table 1).

### DISCUSSION

The *DCC* gene on chromosome 18q is a candidate tumour-suppressor gene that was previously characterized by systematic evaluations of LOH in a series of CRC (Vogelstein et al, 1988, 1989; Fearon et al, 1990). Evidence for a tumour-suppressive function of *DCC* arised from experiments by Klingelhutz et al (1993), who have shown that it can suppress tumorigenesis when exogenously introduced into neoplastic cells lacking *DCC* expression. In addition, the high prevalence of 18q LOH including the *DCC* gene locus and its increasing frequency during colorectal tumour development from early adenomatous stages to carcinoma also argue for a tumour-suppressive function of the *DCC* gene (Vogelstein et al, 1988).

The presence of a functional protein in normal mucosa vs loss in malignant tissue is a prerequisite for the putative tumour-suppressive function of *DCC*. For a panel of gynaecological tumours Enomoto et al (1995) reported reduced or lost expression in endometrial and ovarian malignancies compared with the *DCC* mRNA and protein expression levels in the corresponding normal tissues. We examined DCC protein expression in a panel of matched CRC and normal colonic mucosa. To prevent cross-reactivity we used a monoclonal antibody against the non-homologous intracellular region of *DCC* that was not directed against the high homologous extracellular immunoglobulin- or fibronectin-like domains. As reported by Turley et al (1995), several murine monoclonal antibodies raised against extracellular epitopes of *DCC* failed to detect the DCC protein in normal colonic epithelium. According to the putative tumour-suppressive function of the *DCC* gene produce and to former immunohistochemical results with a polyclonal rabbit anti-DCC antibody raised against the intracellular domain (Hedrick et al, 1994), an intense immunostaining should be seen in normal colonic mucosa detected by a useful anti-DCC antibody. With 15041A, we found that DCC protein expression is almost always lost in CRC, whereas the adjacent normal mucosa is brightly positive, indicating that tumorigenesis is indeed accompanied by the loss of DCC protein. In a few CRC cases, DCC protein was still detectable, but DCC immunoreactivity was mostly restricted to distinct areas. It will be of interest to determine the *DCC* expression in primary and metastatic tumours in these cases. Furthermore, the lower expression in only moderately or poorly differentiated CRC supports the hypothesis that the loss of DCC protein may account for the progression of colorectal tumorigenesis.

The aim of this study was to establish a DCC expression assay for routine clinical practice. Therefore, the tissue preparation procedure had to be a simple macroscopical dissection. Although we kept the contamination of our tumour specimens with normal colonic mucosa cells as low as possible, the frequencies of LOH or lost mRNA expression detected by PCR may be underestimated. We demonstrated LOH in 2 out of 12 informative cases, which is not as frequent as usually reported for colorectal carcinoma, although the published frequencies range widely from 20% to 70% (Vogelstein et al, 1988; Sasaki et al, 1989). Compared with other studies using only a few polymorphic markers or only one PCR based LOH assay (Sasaki et al, 1989; Iino et al, 1993; Iacopetta et al, 1994), our LOH frequency obtained from 16 matched tissue samples after macroscopical dissection by PCR seems quite likely. Even if we cannot exclude that some of the RT-PCR-positive tumours would be *DCC* mRNA negative when tissue had been prepared by time-consuming microdissection, our *DCC* mRNA results are in good agreement with the literature (Itoh et al, 1993). Presently, mainly 18q LOH is used in prognostic assessments. We suggest that the measurement of DCC protein by immunohistochemistry should be introduced in the clinicopathological routine as an additional or alternative prognostic marker. It is easy to perform, not influenced by adjacent normal tissue and only needs small amounts of material.

Homozygous *DCC* gene deletions are restricted to a few published cases (Fearon et al, 1990; Murty et al, 1994) and were not seen in the present study. Typically, the inactivation of a tumour-suppressor gene involves the allelic deletion of one of the two parental alleles, detectable as LOH and according to Knudson (1985) this unmasks a recessive mutation in the remaining allele. A few somatically acquired point mutations in the *DCC* gene generating a novel splice acceptor site or nonsense or missense mutations in the coding sequence as well as insertional mutagenesis in some CRC cases have been reported (Fearon et al, 1990; Miyake et al, 1994). But without any evidence for a general molecular mechanism leading to the inactivation of the *DCC* allele in CRC, the analysis of additional mutations should be pursued. Therefore, we analysed the loss of the wild-type codon 201 in CRC and adjacent normal mucosa. Miyake et al (1994) had previously identified this mutation as a 'polymorphic change' in a single case of oesophageal squamous cell carcinoma and its adjacent normal mucosa but without any data about the codon 201 sequence in PBLs or other somatic cells outside of the oesophageal mucosa. Our data indicate that the C to G transition of the first base in codon 201 is indeed a

 © Cancer Research Campaign 1998

real mutation because all the three PBL samples analysed showed completely digestible wild-type codons 201, whereas the pattern of the *SalI* restriction hydrolysis was already different in two cases of normal colorectal mucosa compared with the corresponding PBLs. Honsako et al (1994) have found that the loss of wild-type codon 201 is significantly increased in invasive colorectal carcinomas and carcinomas with distant metastasis. Thus, the epidemiological data of 18q LOH (Kern et al, 1989; Iino et al, 1994) as well as the results of the codon 201 study (Honsako et al, 1994) appear to be predictive indicators for local invasion and distant metastasis in CRC. As shown by Jen et al (1995) 18q LOH has strong prognostic value for the outcome of CRC patients. To prove that *DCC* has a tumour-suppressive function in colorectal tumorigenesis the prognostic markers 18q LOH and loss of wild-type codon 201 have to be associated with reduced or absent expression of the *DCC* gene product in CRC. In fact, we could show in the present study that the *DCC* protein is not expressed in cases with proven LOH. In particular, *DCC* immunoreactivity was negative in 89% (eight out of nine) of the tumours with loss in both 201 wild-type codons whereas at least one wild-type codon 201 was present in 80% (four out of five) of the tumours with positive immunoreactivity (Table 2). We speculate that loss of both wild-type codons 201 might interfere with the *DCC* transcription or translation. Although our data suggest a correlation between codon 201 and DCC protein expression, we do not believe in loss of wild-type codon 201 as the only cause for inactivation of the remaining allele. Therefore, a situation with no detectable wild-type codon 201 does not lead inevitably to a loss of DCC protein expression (patient UZ, Table 1). In addition, we found that the loss of the wild-type codon 201 is not restricted to CRC. It also occurs with high frequency in the adjacent normal mucosa, resulting in a heterozygous allelotype with one wild-type and one mutant codon. Upon malignant transformation, 53% of the tumours accumulate an additional loss of the retained wild-type codon, thus exhibiting a mutant phenotype with no wild-type codon 201. We speculate that the loss of the first wild-type codon 201 could be an early premalignant event in the colorectal adenoma–carcinoma–sequence. In a further study, it will be of special interest to evaluate the codon 201 status in normal colonic mucosa without any evidence for adjacent CRC and to analyse the biochemical importance of this missense mutation in exon 3 related to the inactivation of a remaining *DCC* allele.

### ACKNOWLEDGEMENT

This study was supported by Grant Di 245/4-1 from the Deutsche Forschungsgemeinschaft.

### ADDENDUM

After submission of this manuscript, an immunohistochemical study correlating the DCC protein expression and the prognosis in colorectal cancer has been published (Shibata, D et al, 1996. *N Engl J Med* **335**: 1727–1732), confirming our thesis that immunohistochemical detection of the DCC protein expression might be a useful clinicopathological marker.

### REFERENCES

Cho KR and Fearon ER (1995) *DCC*: linking tumor suppressor genes and altered cell surface interactions in cancer? *Curr Opin Genet Dev* **5**: 72–78

Cho KR, Oliner JD, Simons JW, Hedrick L, Fearon ER, Preisinger AC, Hedge P, Silverman GA and Vogelstein B (1994) The *DCC* gene: structural analysis and mutations in colorectal carcinomas. *Genomics* **19**: 525–531

Chomczynski P and Sacchi N (1987) Single-step method of RNA isolation by acid guanidinium thiocyanate–phenol–chloroform extraction. *Anal Biochem* **162**: 156–159

Chuong C-M, Jiang T-X, Yin E and Widelitz RB (1994) *cDCC* (chicken homologue to a gene deleted in colorectal carcinoma) is an epithelial adhesion molecule expressed in the basal cells and involved in epithelial–mesenchymal interaction. *Dev Biol* **164**: 383–397

Dippold WG, Dienes HR, Knuth A and Meyer Zum Büschenfelde K-H (1985) Immunohistochemical localization of ganglioside GD3 in human malignant melanoma, epithelial tumors and normal tissues. *Cancer Res* **45**: 3699–3705

Edelman G and Crossin K (1991) Cell adhesion molecules: implications for a molecular histology. *Annu Rev Biochem* **60**: 155–190

Enomoto T, Fujita M, Cheng C, Nakashima R, Ozaki M, Inoue M and Nomura T (1995) Loss of expression and loss of heterozygositiy in the *DCC* gene in neoplasms of the human female reproductive tract. *Br J Cancer* **71**: 462–467

Fearon ER, Cho KR, Nigro JM, Kern SE, Simons JW, Ruppert JM, Hamilton SR, Preisinger AC, Thomas G, Kinzler KW and Vogelstein B (1990) Identification of a chromosome 18q gene that is altered in colorectal cancers. *Science* **247**: 49–56

Hahn SA, Schutte M, Hoque ATMS, Moskaluk CA, Dacosta LT, Rozenblum E, Weinstein CL, Fischer A, Yeo CJ, Hruban RH and Kern SE (1996) *DPC4*, a candidate tumor suppressor gene at human chromosome 18q21.1. *Science* **271**: 350–353

Hedrick L, Cho KR, Fearon ER, Wu T-C, Kinzler KW and Vogelstein B (1994) The *DCC* gene product in cellular differentiation and colorectal tumorigenesis. *Genes Dev* **8**: 1174–1183

Honsako Y, Aoyama N, Futamis S, Tamura T, Morimoto S, Nakashima T, Ohmoto A, Ohamo H, Miyamoto M, Kasuga M, Fujimori T and Maeda S (1994) Codon $201^{Gly}$ in *DCC* gene relates to invasive colorectal carcinoma and its distant metastasis. *Gastroenterology* **106**: A394

Huang Y, Boynton RF, Blount PL, Silverstein RJ, Yin J, Tong Y, McDaniel TK, Newkirk C, Resau JH, Sridhara R, Reid BJ and Meltzer SJ (1992) Loss of heterozygosity involves multiple tumor suppressor genes in human esophageal cancers. *Cancer Res* **52**: 6525–6530

Iacopetta B, Digrandi S, Dix B, Haig C, Soong R and House A. (1994) Loss of heterozygosity of tumour suppressor gene loci in human colorectal carcinoma. *Eur J Cancer* **30A**: 664–670

Iino H, Fukayama M, Maeda Y, Koike M, Mori T, Takahashi T, Kikuchi-Yanoshita R, Miyaki M, Mizuno S and Watanabe S (1994) Molecular genetics for clinical management of colorectal carcinoma. *Cancer* **73**: 1324–1331

Itoh F, Hinoda Y, Ohe M, Ohe Y, Ban T, Endo T, Imai K and Yachi A (1993) Decreased expression of *DCC* mRNA in human colorectal cancers. *Int J Cancer* **53**: 260–263

Jen J, Kim H, Piantadosi S, Liu Z-F, Levitt RC, Sistonen P, Kinzler KW, Vogelstein B and Hamiltion SR (1994) Allelic loss of chromosome 18q and prognosis in colorectal cancer. *N Engl J Med* **331**: 213–221

Keino-Masu K, Masu M, Hinck L, Leonardo ED, Chan SS-Y, Culotti JG and Tessier-Lavigne M (1996) Deleted in colorectal cancer (*DCC*) encodes a netrin receptor. *Cell* **87**: 175–185

Kern SE, Fearon ER, Tersmette K, Enterline J, Leppert M, Nakamura Y, White R, Vogelstein B and Hamilton SR (1989) Allelic loss in colorectal carcinomas. *JAMA* **261**: 3099–3103

Klingelhutz AJ, Smith PP, Garrett LR and McDougall JK (1993) Alterations of the *DCC*-tumor suppressor gene in tumorigenic Hpv-18 immortalizd human keratinocytes transformed by nitrosomethylurea. *Oncogene* **8**: 95–99

Knudson A (1985) Hereditary cancer, oncogenes, and antioncogenes. *Cancer Res* **45**: 1437–1443

Miyake S, Nagai K, Yoshino K, Oto M, Endo M and Yuasa Y (1994) Point mutations and allelic deletion of tumor suppressor gene *DCC* in human esophageal squamous cell carcinomas and their relation to metastasis. *Cancer Res* **54**: 3007–3010

Murty VVVS, LI R-G, Houldsworth J, Bronson DL, Reuter VE, Bosl GJ and Chaganti RSK (1994) Frequent allelic deletions and loss of expression characterize the *DCC* gene in male germ cell tumors. *Oncogene* **9**: 3227–3231

Ponte P, Sun-Yu N, Engel J, Gunning P and Kedes L (1984) Evolutionary conservation in the untranslated regions of actin mRNAs: DNA sequence of a human beta-actin cDNA. *Nucleic Acids Res* **12**: 1687–1696

Reale MA, Hu G, Zafar AI, Getzenberg RH, Levine SM and Fearon ER (1994) Expression and alternative splicing of the deleted in colorectal cancer (*DCC*) gene in normal and malignant tissues. *Cancer Res* **54**: 4493–4501

© Cancer Research Campaign 1998

Sasaki M, Okamoto M, Sato C, Sugio K, Soejima J-I, Iwama T, Ikeuchi T, Tonomura A, Miyaki M and Sasazuki T (1989) Loss of constitutional heterozygosity in colorectal tumors from patients with familial polyposis coli and those with nonpolyposis colorectal carcinoma. *Cancer Res* **49**: 4402–4406

Tanaka K, Oshimura M, Kikuchi R, Seki M, Hayashi T and Miyaki M (1991) Suppression of tumorigenicity in human colon carcinoma cells by introduction of normal chromosome 5 or 18. *Nature* **349**: 340–342

Thiagalingam S, Lengauer C, Leach FS, Schutte M, Hahn SA, Overhauser J, Willson JKV, Markowitz S, Hamilton SR, Kern SE, Kinzler KW and Vogelstein B (1996) Evaluation of candidate tumour suppressor genes on chromosome 18 in colorectal cancers. *Nature Genet* **13**: 343–346

Turley H, Pezzella F, Kocialkowski S, Comley M, Kaklamanis L, Fawcett J, Simmons D, Harris AL and Gatter KC (1995) The distribution of the deleted in colon cancer (*DCC*) protein in human tissues. *Cancer Res* **55**: 5628–5631

Vogelstein B, Fearon ER, Hamilton SR, Kern SE, Preisinger AC, Leppert M, Nakamura Y, White R, Smits AMM and Bos JL (1988) Genetic alterations during colorectal tumor development. *N Engl J Med* **319**: 525–532

Vogelstein B, Fearon ER, Kern SE, Hamilton SR, Preisinger AC, Nakamura Y and White R (1989) Allelotype of colorectal carcinomas. *Science* **244**: 207–211

*© Cancer Research Campaign 1998*